# Investigating the Feasibility of Child Mortality Surveillance With Postmortem Tissue Sampling: Generating Constructs and Variables to Strengthen Validity and Reliability in Qualitative Research

**DOI:** 10.1093/cid/ciz564

**Published:** 2019-10-09

**Authors:** Elizabeth O’Mara Sage, Khátia R Munguambe, John Blevins, Rui Guilaze, Baindu Kosia, Maria Maixenchs, Quique Bassat, Inácio Mandomando, Reinhard Kaiser, Ahoua Kone, Amara Jambai, Nellie D Myburgh, Noni Ngwenya, Shabir A Madhi, Ketema Degefa, Caroline Ackley, Robert F Breiman, Pratima L Raghunathan

**Affiliations:** 1 Center for Global Health, Centers for Disease Control and Prevention, Atlanta, Georgia, USA; 2 Centro de Investigação em Saúde de Manhiça, Maputo, Mozambique; 3 Eduardo Mondlane University, Faculty of Medicine, Community Health Department, Maputo, Mozambique; 4 Emory Global Health Institute, Emory University, Atlanta, Georgia, USA; 5 FOCUS 1000, Makeni, Sierra Leone; 6 ISGlobal, Hospital Clinic-Universitat de Barcelona, Barcelona, Spain; 7 Catalan Institution for Research and Advanced Studies (ICREA), Barcelona, Barcelona, Spain; 8 Pediatric Infectious Diseases Unit, Pediatrics Department, Hospital Sant Joan de Déu, University of Barcelona, Barcelona, Spain; 9 Consorcio de Investigacion Biomedica en Red de Epidemiologia y Salud, Spain; 10 Instituto Nacional de Saúde, Ministério de Saúde, Maputo, Mozambique; 11 US Centers for Disease Control and Prevention--Sierra Leone, Freetown, Sierra Leone; 12 Ministry of Health and Sanitation, Sierra Leone; 13 Medical Research Council: Respiratory and Meningeal Pathogens Research Unit, University of the Witwatersrand, Faculty of Health Sciences, Johannesburg, South Africa; 14 Department of Science and Technology/National Research Foundation, Vaccine Preventable Diseases, University of the Witwatersrand, Faculty of Health Sciences, Johannesburg, South Africa; 15 College of Health and Medical Sciences, Haramaya University, Harar, Ethiopia; 16 London School of Hygiene and Tropical Medicine, London, United Kingdom; 17 Department of Infectious Disease Epidemiology, London School of Hygiene and Tropical Medicine, London, United Kingdom

**Keywords:** qualitative research, feasibility, credibility, child mortality surveillance

## Abstract

**Background:**

The Child Health and Mortality Prevention Surveillance (CHAMPS) network aims to generate reliable data on the causes of death among children aged <5 years using all available information, including minimally invasive tissue sampling (MITS). The sensitive nature of MITS inevitably evokes religious, cultural, and ethical questions influencing the feasibility and sustainability of CHAMPS.

**Methods:**

Due to limited behavioral studies related to child MITS, we developed an innovative qualitative methodology to determine the barriers, facilitators, and other factors that affect the implementation and sustainability of CHAMPS surveillance across 7 diverse locations in sub-Saharan Africa and South Asia. We employed a multimethod grounded theory approach and analytical structure based on culturally specific conceptual frameworks. The methodology guided data interpretation and collective analyses confirming how to define dimensions of CHAMPS feasibility within the cultural context of each site while reducing subjectivity and bias in the process of interpretation and reporting.

**Results:**

Findings showed that the approach to gain consent to conduct the MITS procedure involves religious factors associated with timing of burial, use of certain terminology, and methods of transporting the body. Community misperceptions and uncertainties resulted in rumor surveillance and consistency in information sharing. Religious pronouncements, recognition of health priorities, attention to pregnancy, and advancement of child health facilitated community acceptability.

**Conclusions:**

These findings helped formulate program priorities, guided site-specific adaptations in surveillance procedures, and verified inferences drawn from CHAMPS epidemiological and formative research data. Results informed appropriate community sensitization and engagement activities for introducing and sustaining mortality surveillance, including MITS.

## Teaser

This article describes a unique qualitative methodology used to determine the feasibility of conducting minimally invasive tissue sampling (MITS) on children <5 years of age. Results contributed to adaptations in surveillance and MITS clinical procedures, and informed essential community sensitization and engagement activities.

Although progress has been made to reduce childhood mortality worldwide, approximately 5.6 million children aged <5 years (15 000/day) still die every year [1]. Neonatal deaths, in particular, constitute 46% of under-5 mortality with the annual number of stillbirths, a traditionally neglected group for child mortality statistics, being almost equal to the number of neonatal deaths [1, 2]. Diseases that are preventable through cost-effective and basic quality-delivered interventions may cause most of these deaths [3]. However, understanding the true burden and causes of under-5 mortality is challenged by incomplete and poor-quality primary data, current reliance on modeling, lack of standardized data collection processes, approaches, and delays in the dissemination of academic research. In 2015, only 3% of under-5 childhood cause-specific mortality fractions were based on adequate vital registration data, primarily from high-income countries, and more than one-third of the world’s countries have no cause-specific mortality data at all [4, 5]. Therefore, tracking and monitoring specific causes of under-5 mortality is at the forefront of public health.

The Child Health and Mortality Prevention Surveillance (CHAMPS) Network aims to address under-5 childhood mortality by generating accurate, timely, and reliable data on the causes of death in selected sites in 7 diverse countries in sub-Saharan Africa (South Africa, Mali, Mozambique, Kenya, Sierra Leone, Ethiopia) and Asia (Bangladesh). Cause of death are derived from a complex set of data sources, including clinical medical records, verbal autopsies, and a procedure known as minimally invasive tissue sampling (MITS), which uses biopsy needles to obtain postmortem samples for histopathologic, microbiologic, and molecular examination [6]. CHAMPS program objectives differ from those of traditional studies focusing on disease etiology, because MITS is conducted on the body of a recently deceased child. Inevitably, complex religious beliefs, practices, cultural norms, and ethical questions must be addressed to help improve the feasibility of CHAMPS surveillance implementation and to define community engagement strategies to gain acceptance of CHAMPS procedures. This article presents an innovative qualitative formative research methodology developed and implemented by network-wide CHAMPS social and behavioral science (SBS) teams (located at each CHAMPS site) seeking to understand cultural and religious beliefs, norms, and practices that influence the ability to undertake child mortality surveillance.

## Aims of Formative Research in CHAMPS

CHAMPS SBS formative research has focused primarily on (1) examining the different factors that may be associated with the overall feasibility (eg, acceptability, practicality and implementation) of conducting child mortality surveillance, and (2) understanding community-level knowledge, acceptance, support, and approaches for CHAMPS surveillance efforts. In general, studies that focus on feasibility help to determine the appropriateness, relevance, and sustainability of specific research, procedures, and/or interventions. However, in contrast with traditional feasibility studies, CHAMPS SBS formative research employs a multiapproach design to ascertain modifications needed to implement surveillance as a whole and understand the dimensions of community-level acceptability (and nonacceptability) within the context of each CHAMPS site’s culture and social environments. More specifically, CHAMPS SBS formative research generates data on community perceptions of norms and practices that may affect the implementation of child mortality surveillance and/or the promotion of CHAMPS as an initiative striving to improve overall child health among communities.

Another important aim of CHAMPS qualitative formative research focusing on feasibility involves identifying and understanding how to respond to known and unanticipated perceptions, rumors, interests, sigma, concerns, barriers, and opportunities that could arise through CHAMPS activities. In addition, the formative research assesses if pregnancy, birth, postpartum, and newborn care practices facilitate or impede notification of births, stillbirths, and neonatal deaths to better understand birth outcomes. Through this process, CHAMPS is able to provide culturally sensitive recommendations for actions that aim to optimize programmatic feasibility of surveillance system procedures and community engagement.

The methods and findings discussed in this article describe how the CHAMPS network (1) recognized complexities of feasibility studies regarding child mortality surveillance; (2) built the competencies of CHAMPS researchers based on credible qualitative research; and (3) utilized findings to inform context-specific surveillance and community engagement strategies.

## Credibility in CHAMPS Qualitative Data

Qualitative research is often criticized because methods of data collection and analytical procedures are subject to researcher assumptions, influence, and personal opinions [7]. Thus, concepts such as reliability (consistency within the employed analytical procedures) and validity (integrity in applied methods and precision in which results/findings accurately reflect the underlying data) traditionally associated with credibility and quality in quantitative research are inherently challenging to apply to qualitative studies [7, 8]. Qualitative data are narrative and thematically analyzed and theoretical foundations naturally differ among qualitative researcher experience, competence, and cultural backgrounds. Consequently, statistical measures cannot be applied to evaluate the reliability and validity of qualitative research in the same way as in quantitative studies.

CHAMPS recognized the need to produce credible qualitative research to address important cultural sensitivities and religious beliefs, practices, and concerns around child death. We anticipated that qualitative approaches would provide meaning behind negative perceptions of CHAMPS objectives and rumors associated with surveillance methods and clinical procedures. Thus, the information described in the following section on methods details how CHAMPS formative research was designed to apply sound, reliable, and standardized approaches to help improve the general feasibility of surveillance for child deaths and MITS procedures, and address necessary community engagement strategies in settings of high child mortality.

## METHODS

### Practical Uses of Theory to Explore Feasibility

CHAMPS SBS feasibility studies consist of sociological and anthropological approaches, namely, ethnography and phenomenology [9]. Ethnography is an approach seeking to understand how a group of people (ie, community) forms and sustains a “culture” around a specific topic, which may result in a clearer understanding of certain cultural behaviors and practices [10, 11]. This approach allows opportunities to explore cultural and social phenomena (ie, child death) in their broader contexts to understand local meaning and practices (eg, burial, death notification, grief) [9]. In addition, an ethnographic approach prioritizes immersion into the sample population to a greater degree than other theoretical approaches, which presents opportunities to establish trust and partnerships needed to appropriately interpret the concept of feasibility and implement CHAMPS surveillance [12]. As a complimentary method, phenomenology seeks to understand meanings, features, and a sense of a first-hand experience(s) reflective of research interest [13, 14]. As previous studies have focused more on hypothetical responses about the acceptability of conducting MITS, we determined that firsthand experiences from parents who have lost a child, for example, could provide more accurate evidence regarding MITS acceptability [15–17]. This approach enables SBS investigators to move from theoretical impressions about potential factors influencing CHAMPS feasibility into understanding how these factors operate in actuality [9].

Due to limited behavioral studies supported by theoretically sound paradigms related to conducting MITS on children, we determined that a grounded theory approach was the best option for data analysis. In contrast to more traditional theoretical and thematic analyses interested in describing human experience, grounded theory focuses on generating theory (to create/influence program recommendations) from data opposed to examining an existing theory, therefore concentrating on the process of collecting and analyzing data in the absence of a hypothesis [14, 18]. Instead of relying on a preconceived theory to link back to our conclusions, we can conclude that any theoretical premise generated from the analysis is based on findings, resulting in deeper understanding and meaning to our concepts regarding acceptability, practicality, and implementation of mortality surveillance and community engagement.

### Ensuring CHAMPS Work Is Culturally Sensitive and Appropriate

In addition to studying religious beliefs, practices, and cultural norms, CHAMPS SBS formative research considers other key factors that may impact surveillance implementation and community engagement approaches. These factors (variables) include current political and economic conditions, existing disease patterns, health concerns, and environmental factors. From a socioecological perspective, these additional variables are applied to help characterize the perspectives and behaviors at familial, community, and societal levels while ensuring that CHAMPS surveillance and community engagement planning is culturally sensitive and appropriate. Furthermore, the examination of these factors requires intentional, ongoing, respectful partnerships and consistent multidirectional communications with community members and community leaders, including religious, traditional, and political leaders. Such partnerships are essential for establishing the conditions on which trust can be built and strengthened over time [9]. Thus, to solidify and maintain these partnerships, teams comprised of social scientists, anthropologists, field workers engaged in SBS activities, and public health practitioners have been formed at each CHAMPS site. These CHAMPS “SBS teams” also conduct research design modifications and implementation of CHAMPS SBS activities and use formative research findings to inform other components of the CHAMPS program at their respective site (as detailed in the following sections).

### Addressing Credibility in Research Design, Data Collection, Analysis, and Reporting

CHAMPS SBS teams employ systematic, yet flexible qualitative methods in the design and implementation of formative research feasibility studies. Based on the culturally specific conceptual frameworks, data collection standards reflective of ethnography and phenomenology, and a grounded theory approach to analysis, CHAMPS SBS teams plan the specific direction by which the research is undertaken by generating themes from the data rather than examining an existing hypothesis or theory; such an approach follows a more inductive theoretical and analytical framework. An inductive approach argues that theory will emerge from the data in the absence of a theoretical framework that provides insight, guidance, and support to the study [19]. However, the incorporation of consistency, constant comparative standards (multi/peer), culturally adapted research designs, triangulation, and other approaches described below help to recognize common bias and reactivity imposed and reproduced by qualitative researchers to reduce potential threats to qualitative reliability and validity (credibility) [8]. Of note, the aims of initial formative research did not include generalizability or external validity. Separate qualitative meta-analyses are in progress exploring cross-site factors associated with CHAMPS acceptability.

### Ethical Considerations

A generic SBS protocol, “Assessment of community perceptions and the feasibility of conducting child mortality and pregnancy surveillance protocol,” was independently reviewed and approved by the Emory University Institutional Review Board (IRB) (the US Centers for Disease Control and Prevention relied on Emory IRB approval) [9]. Based on this general protocol, each CHAMPS site developed and modified its own site-specific SBS protocol to reflect the context and idiosyncrasies of each site’s culture and research standards. Site SBS protocols were subsequently submitted as separate protocols to the appropriate local ethical review committee(s) with local oversight.

Implementation of rigorous ethical guidelines is critical when planning and conducting research on child mortality. Guidelines help to minimize the risk of potential harm to participants, researchers, and others, and maximize likelihood that risks are outweighed by benefits. Unfortunately, there is a serious disparity in internationally recognized or agreed upon ethical standards and guidelines for researching child death and potential harm inflicted on a parent(s) being questioned about their child’s death [20–22]. Thus, researchers are limited by the lack of resources to help guide and address ethical considerations and possible dilemmas different from that of more traditional qualitative research [22]. There is some evidence that parents of a deceased child will be willing to talk about their experiences of loss under a supportive structure; however, researchers must be prepared to address any challenges or dilemmas resulting from a strong emotional response or inadvertent provocation of distress [23, 24]. In the context of CHAMPS qualitative formative research, data collection must be carried out in the most ethically sound method as possible. Therefore, ethical training and protocols are created to include planned steps and responses required to protect the participant and interviewer if a dilemma were to occur. In addition, findings presented in recommendations and reports are carefully constructed according to realistic capacities and construed to prevent misinterpretation of blaming (eg, parent[s], medical staff, traditional healer) for a child’s death. To this end, formative research reports employ evidence-based standards with a pragmatic approach to theoretical data analysis, interpretation, and reporting.

### Conceptual Frameworks

Conceptual frameworks are derived from time-tested philosophies foundational to investigations on how phenomena occur and the meaning behind them [25]. However, theoretical premises associated with under-5 mortality, including the role of MITS, have not been widely explored. To this end, CHAMPS formative research is based on a common set of objectives providing the bases for site-specific research questions exploring the feasibility of child mortality surveillance and community engagement approaches (see Appendix). From these common objectives, each CHAMPS site builds its own culturally appropriate conceptual framework comprised of (1) defined site-specific constructs reflective of CHAMPS feasibility (eg, acceptability, practicality and implementation) and (2) measurable variables (eg, approaches for parental consent, burial timing and practices for a child, incentives). Each conceptual framework presumes the potential relationships between the constructs and specific variables identified in the study and is used as a “checklist” to help track what has been discovered and what requires further exploration. Thus, the conceptual framework embodies the specific direction by which the formative research is carried out and helps researchers remain focused on the input, process, and output of each investigation.

### Development of Site-specific Conceptual Frameworks and Sampling Strategies

As a first step to foster reliability and validity in CHAMPS qualitative formative research, the development of site-specific conceptual frameworks were essential to promote standardization and consistency. In general, Maxwell describes conceptual frameworks as a “system of concepts, assumptions, expectations, beliefs, and theories that supports and informs the research” (p. 39)[25]. The development of a conceptual framework as an initial stage of research design provided SBS teams an opportunity to document agreed-upon categories for examination, and demonstrate consistency to reassure reliability [26]. Each CHAMPS SBS team built and defined constructs and variables that would lead to discovery of similar phenomena [27]. These initial conceptual frameworks were based on common constructs derived from CHAMPS objectives, previous studies, specific aims of the research, and SBS team consensus [9]. Not surprisingly, all CHAMPS sites agreed upon the same initial constructs to guide their formative research and community engagement strategies; these constructs included acceptability, practicality, and implementation. These 3 guiding constructs also helped to define and interpret findings from Participatory Inquiry Into Community Knowledge of Child Health and Mortality (PICK-CHAMP) workshops, which assess initial levels of alignment and tension between community priorities and CHAMPS objectives. By identifying instances (eg, death practices, consent to MITS, community tensions) that would lead to the exploration of a larger phenomenon (acceptability of MITS), each CHAMPS site generated additional constructs in accordance with their own context and cultural interpretations. Specific variables were then identified to help measure and categorize each defined construct of the conceptual framework. However, since grounded theory is also used more generically “to denote theoretical constructs derived from qualitative analysis of data,” as described throughout Strauss and Corin’s *Basics of Qualitative Research* (pp. 158–175) [28], constructs are consistently revised and new constructs are derived from findings. Thus, the conceptual framework is a fluid and iterative research tool. Table 1 illustrates the descriptive similarities and differences among constructs exploring feasibility in 2 CHAMPS sites, Sierra Leone and Mozambique. These are further compared to how a construct may be generally defined in a more traditional study investigating feasibility. Table 2 lists the different variables identified to measure feasibility of CHAMPS activities according to each construct (acceptability, practicality, and implementation) in Sierra Leone and Mozambique.

**Table 1.** Constructs Used to Examine Feasibility in Sierra Leone and Mozambique

Construct	General Examples	Sierra Leone	Mozambique
Acceptability	Recipients’ reactions to mortality surveillance and how this may benefit or hinder their daily, cultural, and/or religious beliefs and practices	The degree/extent to which an individual agrees or not or responds to CHAMPS activities (eg, mortality surveillance, pregnancy surveillance) in relation to beliefs, attitudes, knowledge, etc	Opinions, perceptions, reactions, and suggestions of the stakeholders regarding CHAMPS activities, influenced by their beliefs, norms, values, practices, and economic factors, time, and distance
Practicality	The extent to which MITS can be performed when resources, time, or other factors are impeded	The beliefs, resources, opportunities, legal, and ethical considerations and approaches that can aid or limit the carrying out of CHAMPS activities	Possibility of carrying out MITS and other mortality and pregnancy surveillance activities taking into account the presence (or absence), alone or in combination, of barriers or difficulties
Implementation	The probability and method in which mortality surveillance is applied and executed as planned	Requirements and approaches that should be considered in the planning and execution of CHAMPS activities	Probability to execute CHAMPS activities according to time, places, and resources planned, taking into account (1) ethical considerations, (2) pregnancy and infant mortality, (3) availability of stakeholders, and (4) external factors (political conditions and lack of resources)
Other		Inclusiveness: cross-cutting collaborations, partnerships, and other broad-based relationships required to promote necessary action to enhance acceptability, practicability, and implementation	

Abbreviations: CHAMPS, Child Health and Mortality Prevention Surveillance; MITS, minimally invasive tissue sampling.

**Table 2.** Variables Measuring the Constructs Listed in the Conceptual Frameworks, Sierra Leone and Mozambique

Construct	Sierra Leone	Mozambique
Acceptability	• Perceptions of preferred community reporters • Awareness of rituals and grieving and the appropriate ways to address these through community engagement • Knowledge about CHAMPS • Perceptions about causes of child death • How a community would perceive CHAMPS • Desire/willingness to gain knowledge of the cause of death and consent to CHAMPS activities • Gender-specific beliefs and attitudes regarding different stages of pregnancy and delivery • Community perceptions about the capacity and quality of ANC and delivery • Processes/procedures for preparing the body (after death) for burial • Facility procedures that occur after the death of a stillbirth, neonate (0–28 days old), 1 year old, etc	• Beliefs about child death, corpse, religion, traditions, and confidentiality • Desire/willingness to consent and gain knowledge of the cause of death • Relevant cultural practices • Rituals and grieving • Stigmas associated with stillbirths and neonatal deaths • Beliefs about early pregnancy loss, stillbirth, and neonatal death • Community understanding and acceptance of public health initiatives such as CHAMPS • Community perceptions about the capacity and quality of ANC and delivery • How to communicate the value of child mortality surveillance and MITS • Clinician perceptions • Approaches for identifying the key community stakeholders that should be involved in examining community entry • How to monitor “acceptability” and address rumor control
Practicality	• Rituals and grieving (age, gender, and community) • Stigmas associated with stillbirths and neonatal deaths • Barriers associated with access to care involving ANC, health facility delivery and newborn care, and postnatal care • Legal issues that may impact CHAMPS activities • Facility capacities in pregnancy dating, skilled birth attendant coverage, and postpartum and newborn exams	• Beliefs about the incentives that may play a role in CHAMPS activities, history of incentives in target communities, and effect on participants’ perceptions • Collaborations and relationships with ministries of health and other relevant government and nongovernmental agencies • Patterns associated with pregnancy notification, care-seeking behaviors, delivery planning including location of delivery and desired birth attendants, birth notification, and postpartum practices • Barriers associated with access to care involving ANC, health facility delivery and newborn care, and postnatal care • Provider perceptions regarding ANC policies, preferences, and improvements related to ANC and postnatal and newborn care • Methodologies for identifying the benefits of CHAMPS activities on the existing clinical and laboratory infrastructure/services in the community as a value-added outcome • Awareness of rituals and grieving and the appropriate ways to address them in community engagement
Implementation	• Training needs for those involved in CHAMPS activities • Approaches for identifying the key community stakeholders that should be involved in examining community entry • Roles of governmental authorities	• Requirements for health systems to accept and participate in child mortality surveillance utilizing MITS, including reluctance and competing priorities • Facility capacities in pregnancy dating, skilled birth attendant coverage, and postpartum and newborn examinations • Issues that impact CHAMPS activities in the countries where the surveillance activities will be implemented • Role of governmental authorities (eg, ministries of health) in CHAMPS activities
Inclusiveness	• Approaches for identifying the key community stakeholders that should be involved in examining community entry • Collaborations and relationships with ministries of health and other relevant government and nongovernmental agencies • Health worker concerns	

Abbreviations: ANC, antenatal care; CHAMPS, Child Health and Mortality Prevention Surveillance; MITS, minimally invasive tissue sampling.

To reduce the possibility of bias due to investigator selection of specific informants who are more likely to be supportive of the program, the initial research design involved development of a strategic sampling framework. Each initial framework consists of a defined, diverse number of key informants, representatives of community groups, and/or individuals who could best describe, discuss, and explain their perspectives and experiences concerning the phenomenon (construct) being explored. Examples included, but were not limited to:

- Knowledgeable leaders in the community (notables, village chiefs, elders, heads of nongovernmental organizations, and local political and traditional authorities)
- Community-level healthcare providers (traditional healers, matrons, physicians, social workers, and traditional birth attendants)
- Professionals involved in proceedings related to death (mortuary attendants, body preparers, undertakers)
- Religious leaders (representatives of world religious traditions and local belief systems)
- Local community members (parents and/or next of kin of children under 5, parents who have lost a child, child caregivers, pregnant women)

We learned that sampling should be representative of target communities as a whole because members and leaders of one community may either be involved with what is being explored (ie, consent to MITS) or affected by it, or could influence others within or outside these communities. Because formative research activities are expected to start prior to and throughout program implementation, sampling was thoughtful, flexible, and strategic to ensure catchment area representativeness and reduce the chance of bias in reporting [25]. We worked closely with demographic surveillance system teams at country sites where they previously existed, and through relationships established during community entry activities, including PICK-CHAMP workshops. Furthermore, the predetermined constructs and variables helped identify the initial types of participants (not the actual individual) and the iterative process modified subsequent sample populations driven by the data (theoretical sampling).

### Data Collection

Consistent with the common premise of grounded theory analysis, we undertook meticulous planning and attention to each step of the research process to reflect the cultural and societal diversities of each community where CHAMPS is implemented. In other words, we sought methods that would best represent data triangulation and representation of the “community at-large” rather than just on the level of the participating “individual” (see sampling in previous section). To meet this standard, various fundamental data collection methods were employed and combined, including key informant interviews, focus group discussions, semi-structured interviews, and observation techniques. Participants for semi-structured interviews were chosen to reflect the cultural group they were chosen to represent with the assumption that the subject’s perspectives yield insights into the larger perspectives of the group as a whole. By interviewing a range of subjects representative of a target group, the general view of that group was ascertained. Subjects for key informant interviews were chosen because their perspectives were influential in shaping community norms and opinions. This influence may be the result of the subject’s formal position (eg, a health official or a religious leader); however, the influence may be informal as a result of social relationships within the community. Key informants were chosen to yield insights on opinions that could be influential for the community as a whole. Focus groups were convened to yield participants’ perspectives on more complex phenomena (eg, religious beliefs about the meaning of death) because the conversational nature of the interview in a group setting would influence each individual participant to reflect more fully on their own perspectives and provide more information as they listened to and responded to the perspectives of other participants. SBS staff in each site established targets on the number of semi-structured interviews, key informant interviews, and focus group discussions that would be carried out to yield information on the constructs of their conceptual frameworks.

SBS staff also identified a number of community practices related to the death of children that they would observe in real time. Such practices included community and familial religious rituals that provided a basis by which communities made meaning of childhood deaths. Such meanings may not be ascertained in interviews or focus group discussions because they were not always verbally articulated; nonetheless, such meanings influenced community perceptions and norms. Through observation, the SBS teams were able to identify rituals that carried meaning and then de-brief with community members to articulate those meanings.

All interviews were recorded and transcribed to carry out the conceptual analysis. SBS staff who observed community or familial events completed field notes during or immediately after the events and de-briefed on those events with SBS colleagues to determine which rituals impacted perceptions of childhood death.

In addition, CHAMPS SBS teams involved multiple researchers in both the process and the product of research including the development of the data collection tools, participant recruitment, equipment preparation, logistics, etc. To further maintain credibility and objectivity, researchers received training on how to be reflexive, reflective, and aware of influences on their internal and external responses, relationship with participants, and the research topic. For example, the interviewers practiced common techniques to reduce subjectivity by identifying and limiting personal influences through fostering neutrality, interest, positivity, and respect.

Another important aspect of generating credibility involves assessing researcher assumptions prior to and throughout data collection and analysis. Researcher assumptions are generally unconscious thoughts and underlying beliefs that may drive behavior and influence assessment of a response or perception. Researcher assumptions may also influence participant responses by inadvertently constraining values, time, and boundaries. Since “researchers seek to provide an overview whereas participants have individual concerns, and this can result in apparently discrepant accounts” (Barbour p. 1117) [29], they must truly scrutinize the “complex interplay of own personal biography, power and status, interactions with participants, and the written word [reporting findings]” (Rossman p. 95) [10].

Application of these different data collection approaches help verify the nature and integrity of inferences drawn from the diverse data collected. These combined qualitative methods have been implemented in a staged, cyclical approach to facilitate data triangulation needed to validate findings helpful in the modification of CHAMPS implementation, recognition of program priorities and community partnerships, and comprehensive community sensitization and engagement activities. This approach also facilitated simultaneous data collection and data analysis to continuously modify and improve data collection instruments while capturing and learning from emerging themes.

### Data Analysis

Grounded theory guides researchers to code and categorize data systematically, yet reflectively, to recognize pertinent patterns in the information gathered [30]. As interrelationships between the categorized data (themes) are developed and strengthened through this process, the conceptual foundation/explanation is derived. Examining feasibility involves (1) collecting the data in iterative stages; (2) conducting theoretical sampling; (3) coding the data; (4) assessing relationships between the codes, variables, and constructs; and (5) conducting interpretations linked to the evolving conceptual and/or theoretical foundations of the study. Consistent with this approach, conceptual frameworks developed by the site SBS teams guided the research questions, aligned findings, and fostered new understanding of the variables and constructs that have emerged from the data. CHAMPS investigators conducted in-depth analysis rooted in grounded theory by following these analytic steps:

1. Create a plan to analyze the data
2. Evaluate the quality of the data
3. Closely review and prepare the data for coding
4. Develop a clear understanding of possible codes
5. Begin coding while developing, refining, and defining codes (nodes)
6. Keep analytic memos
7. Describe and compare codes
8. Categorize and conceptualize
9. Triangulate findings
10. Report formative research findings and determine next steps

To address reliability and reduce bias in analytical interpretation, standardization of transcribing procedures requires a minimum of 2 team members involved in the process of a single transcription, of which one leads the actual transcription process and the other is responsible for quality assurances (re-listening to the recorded information and comparing it to the text, modifying the text accordingly). Group analysis was conducted by coding instances to similar categories, interpreting the data, and documenting the procedures; this type of analysis helped to clarify assumptions and allow for constant and standardized comparative assimilation of codes and emerging themes in the data [31, 32].

### Reporting

High quality of reports is essential and dependent upon (1) decisions about which data or analyses to omit (data reduction) and (2) how much description to include [14]. In general, data reduction is carried out by first deciding how much description is necessary. Well-developed and concise descriptions help illustrate and clarify perceptions and general representation of a feasibility study. In CHAMPS formative research reports, for example, interpretation of data has been based on how individual perspectives connect to both predefined (conceptual framework) and emerging (data-driven) themes describing the facilitators and barriers associated with the feasibility of CHAMPS surveillance and community engagement activities. Constant connections and descriptions of the relationships between participant perceptions, variables, and constructs has helped us ascertain emerging themes. Furthermore, descriptions have not been inconsequential or subjective, but significant and backed by supporting evidence (eg, data that can traceable back to the source). SBS team members have documented variations of the data in their reports, enabling them to verify credibility of findings. For example, SBS researchers have explored whether patterns in themes are clear, strongly supported, or only suggested, and have noted this when generating actionable recommendations, discussion, and/or conclusions.

## RESULTS

### General Findings That Support Cross-site Credibility in CHAMPS Formative Research

As anticipated, preliminary formative research reports yielded recommendations for how to implement surveillance, yet results could not necessarily be generalized because of the rich cultural diversities across the CHAMPS sites. However, an informal comparison of formative research findings indicated reasonable internal validity and enough reliability to produce cross-site best practices and modifications for overall MITS and community engagement planning, implementation, and sustainability. For example, findings (as expected) showed that the approach to MITS consent should involve close attention and adherence to specific religious and cultural factors associated with timing of death to burial and the type of burial is associated with differences in age at death (ie, stillbirth, neonate, or child). Initial findings also informed appropriate community sensitization and engagement activities critical to the implementation of mortality surveillance, such as the necessity to involve religious leaders in the family decision-making process so they can address any questions related to religious doctrines and assure families that their participation in mortality surveillance does not violate religious tenets. Moreover, communities commonly expected that CHAMPS would partner with agencies that provide child health interventions. Findings highlighted community misperceptions and uncertainties (fears, suspicions, and reservations) about CHAMPS, emphasizing the need for rumor surveillance programs and consistent communications and information sharing across the CHAMPS network. Other findings indicated that religious pronouncements, recognition of health priorities, attention to healthy pregnancy, and advancement of child health facilitates general community acceptability.

### Examples of How SBS Findings Have Directly Led to Site-specific Adaptations

Each CHAMPS site has conducted SBS feasibility studies following the same standards as presented above. Below are examples of findings from the SBS teams in Mozambique and Sierra Leone.

#### Mozambique

SBS findings revealed the need to involve local authorities in death notification, such as the secretaries of the neighborhoods. A person with experience in health counseling should perform informed consent for MITS. The social welfare services should be engaged in transporting the body from hospital to the household after the MITS procedure to reduce additional financial burdens endured by the family.

Findings also revealed the necessity of keeping communities consistently informed about the progress of CHAMPS and MITS through routine community meetings, and indicated that a CHAMPS clinical team guided and led by a religious leader should provide cause of death feedback to the family and community. Furthermore, SBS also explored community member perceptions about the most common disease(s) that result in child deaths. This information has been incorporated into epidemiological and program planning in the Manhica site.

#### Sierra Leone

SBS findings informed the most appropriate method and culturally appropriate language to introduce the CHAMPS program and objectives to communities in the Bombali District of Sierra Leone. In addition, findings informed revisions of the verbal and social autopsy tools on specific terminologies that are both locally understood and culturally sensitive. The 2014–2015 Ebola outbreak in Sierra Leone involved public health actions that interfered with important traditional burial practices, triggering understandable misconceptions, fears, and concerns about how the public health system deals with death. To this end, Sierra Leone SBS findings helped to clarify the types of community entry and sensitization efforts required in the proposed CHAMPS catchment area before any surveillance activities commenced.

SBS findings in Sierra Leone have also led to the recruitment and training of key community representatives who work with the surveillance team to set up a system that reports both community and facility deaths. Similarly, the SBS work has informed standard operating procedures regarding how, to whom, and when MITS consent should be administered. Also, a decision was made to offer transport support for all eligible deaths in health facilities (and ideally, for all eligible community deaths) that are approached for MITS regardless of whether or not they consent. Last, as part of the MITS standard operating procedure, a member of the CHAMPS team should be present with family after MITS has been conducted to show solidarity, support, and respect for the dead as is the cultural norm.

## DISCUSSION

The approaches to reducing threats to credibility presented in this article converge on consistency in research design, researcher competencies, and standardization of methodological approaches. However, given the differing opinions on what constitutes “credible” qualitative research, there is no consensus for assessing the different approaches for qualitative research design and implementation. In general, leading schools of thought include the work of Dixon-Woods et al, “which emphasizes on methodology” (p. 325) [33], that led to the 10 questions for the Critical Appraisal Skills Program checklist for qualitative studies, and that of Lincoln, “which stresses the rigor of interpretation of results” [34–36]. Conversely, regardless of technical fixes, published procedures or checklists, it seems that standardization of common approaches exploring similar phenomena combined with systematic and thoughtfully conducted methods and analysis indeed strengthen the unique quality and contributions of qualitative studies exploring complex and sensitive issues such as those prompted by CHAMPS.

### Limitations of Using Grounded Theory in CHAMPS Formative Research

Although findings generated from grounded theory analysis helped form program priorities and site-specific adaptations in surveillance procedures, this approach was associated with some limitations. For example, since grounded theory is the study of a set of roughly defined, but common constructs, interpretation of data collected requires a significant amount of analysis, imposing delays in presentation of recommendations for surveillance implementation. While the relationships between constructs help to identify the theoretical explanation of action(s) needed to resolve main concerns and misperceptions of communities, the essential meaning of each variable and the collective relationships between variables and constructs are difficult to confirm given the lack of baseline data and previous studies on under-5 mortality surveillance involving MITS.

The current CHAMPS formative research methodology does not employ methods complementary to traditional surveys and epidemiological approaches. The exploration of several variables at the same time typically contributes to fuller clarification of feasibility; however, we found interpretation to be an iterative process deserving of a longitudinal approach to comprehensively characterize the complexities and dimensions of CHAMPS feasibility.

The intricacies, variety, and diversity of behaviors, attitudes, and beliefs about child health and mortality are based on a minimal representation of participant responses and thus are not generalizable to a population outside of the CHAMPS catchment areas. Instead, outcomes are based on the interaction of “variables” and “constructs” opposed to a quantitative model with main effects. Other limitations associated with the objectives of CHAMPS formative research involve (but are not limited to) the complexities of behaviors that may involve, for example, one-time behavior vs lifestyle and culturally appropriate behaviors; tension between researchers’ needs and community goals; and/or, tension between the search for theoretical understanding and practical needs of the community involving usefulness, social and cultural naiveté, and/or conflict of values or priorities.

### Recommended Future Opportunities for This Type of Methodology

The findings presented previously confirm simple approaches that can be incorporated into the qualitative research process to increase credibility. However, it is important for qualitative research focused on complex and sensitive topics to seek deeper inquiry of meaning and dimensions of the constructs under examination. For example, it would be valuable to examine other variables that may influence consent such as use of a grief counselor, approaches to single parent consent, and/or timing of consent for deaths that occur in the community. To this end, perhaps closer examination of traditional institutions that influence representativeness (eg, reaching recalcitrant or hidden populations), partners, or timeliness on a more socioecological level would be warranted to encourage cross-culture generalization. In addition, acceptability could be closely monitored at various levels (eg, individual, family, community, and social structural). Similar mixed method approaches that employ evaluations of community engagement activities with qualitative interviews could monitor acceptability among community stakeholders and relevant standards leading to positive actions and/or interventions toward child health and community trust needed to optimize surveillance implementation.

In conclusion, initially employing a rigorous qualitative methodology for CHAMPS formative research was vital to creating a foundation for addressing community perceptions about surveillance (including MITS), to make actionable recommendations that aim to optimize the feasibility and sustainability of surveillance system procedures and community engagement strategies. Furthermore, the methods described in this article fostered strong and independent SBS capacities at each site and also helped to shape how SBS teams use their evidence-based data to strategically manage and promote facilitators and respond to barriers, misperceptions, and/or ethical dilemmas encountered by the implementation of CHAMPS mortality surveillance.

## Notes

***Acknowledgments.*** The authors are grateful to the following individuals for their contributions to the development and implementation of Child Health and Mortality Prevention Surveillance (CHAMPS) social and behavioral formative research: Professor Samba Sow, Dr Adama Mamby Keita, Dr Tieman Diarra, Dr Issa Fofana, and Mr Kounandji Diarra, Centre pour le Développement des Vaccins du Mali, Bamako; Dr Milagritos D. Tapia, Center for Vaccine Development, University of Maryland, Baltimore; Dr Anna Seale, London School of Hygiene and Tropical Medicine in Harar, Ethiopia; Dr Nega Assefa, University of Haramaya in Harar, Ethiopia; Shahana Parveen, Saiful Islam, Muhammad Faruqe Hussain, Dr Emily Gurley, Dr Shams El Arifeen, Dr Sanwarul Bari, icddr,b in Dhaka, Bangladesh; Peter Otieno, Kenya Medical Research Institute in Kisumu; and Dr Emily Zielinski-Gutierrez, Centers for Disease Control and Prevention (CDC) in Nairobi, Kenya. ISGlobal is a member of the Centres de Recerca de Catalunya Programme, Generalitat de Catalunya (http://cerca.cat/en/suma/). Centro de Investigação em Saúde de Manhiça is supported by the government of Mozambique and the Spanish Agency for International Development.

***Financial support.*** This work was supported by the Bill & Melinda Gates Foundation [OPP1126780].

***Supplement sponsorship. *** This supplement is sponsored by the Emory Global Health Institute and the Bill & Melinda Gates Foundation.

***Potential conflicts of interest.*** S. A. M. has received grants from the Bill & Melinda Gates Foundation, Pfizer, Glaxo Smith Kline, Biovac, Novavax, and Sanofi Pasteur; and honoraria from The Bill & Melinda Gates Foundation. All other authors report no potential conflicts of interest. All authors have submitted the ICMJE Form for Disclosure of Potential Conflicts of Interest. Conflicts that the editors consider relevant to the content of the manuscript have been disclosed.

## **APPENDIX:** CHAMPS Sociobehavioral Science Rationale And General Objectives

Original rationale for formative research:

To understand specific cultural, religious, and sociobehavioral factors that may increase or decrease the feasibility of conducting minimally invasive tissue sampling on children aged <5 years, and the factors that may influence pregnancy and neonatal outcomes.

1. To describe cultural, social, and religious norms, rituals, and practices involving the death of a child (stillbirth, newborn, infant, and child) and pregnancy, pregnancy loss, birth, postpartum, newborn, and care
2. To examine facilitators and barriers related to under-5 mortality surveillance, both theoretically and in actuality
3. To examine facilitators and barriers in identifying stillbirth and neonatal deaths
4. To determine factors affecting acceptability of under-5 mortality surveillance, including motivators and barriers, by the relatives of the deceased child, community leaders, and other community members involved
5. To inform tools and approaches for ongoing Child Health and Mortality Prevention Surveillance (CHAMPS) activities and to adapt approaches as community awareness and perceptions evolve and relationships with communities are strengthened
6. To assess the success of community engagement efforts and identify approaches aimed to increase both general practicality and implementation of under-5 mortality surveillance, and acceptance by parents who are requested to participate in CHAMPS activities
